# A dedicated surveillance network for congenital toxoplasmosis in Greece, 2006-2009: assessment of the results

**DOI:** 10.1186/1471-2458-12-1019

**Published:** 2012-11-22

**Authors:** Maria Aptouramani, Maria Theodoridou, George Syrogiannopoulos, Andreas Mentis, Vasiliki Papaevangelou, Katerina Gaitana, Alexandros Daponte, Christos Hadjichristodoulou

**Affiliations:** 1 Greece-Cyprus Pediatric Surveillance Unit (GCPSU), Laboratory of Hygiene and Epidemiology, Faculty of Medicine, University of Thessaly, Larissa, Greece; 2 1st Department of Pediatrics, Faculty of Medicine; Greece-Cyprus Pediatric Surveillance Unit (GCPSU), University of Athens, Athens, Greece; 3 Department of Pediatrics, Faculty of Medicine, University of Thessaly, Larissa, Greece; 4 Pasteur Hellenic Institute, Athens, Greece; 5 2nd Department of Pediatrics, Faculty of Medicine, University of Athens, Athens, Greece; 6 Department of Obstetrics, Faculty of Medicine, University of Thessaly, Larissa, Greece

**Keywords:** Congenital Toxoplasmosis, Surveillance Network, Greece

## Abstract

**Background:**

Toxoplasmosis is caused by infection with the protozoan parasite *Toxoplasma gondii*. Acute infections in pregnant women may be transmitted to the fetus and cause severe illness. The purpose of this study was to establish a dedicated surveillance network (DSN) for congenital toxoplasmosis (CT) in Greece, in order to assess the birth prevalence of CT.

**Methods:**

A DSN of thirty clinicians was established for reporting CT cases from hospitals throughout Greece. The clinicians were selected on the basis that there was a high possibility the suspected cases would be referred to them from district hospitals or private clinics. Suspected cases of CT were reported on a monthly basis with a zero reporting card during a surveillance period from April 2006 to December 2009. A questionnaire was sent for any suspected case to record information including demographic parameters, clinical signs and symptoms and laboratory results. Serological and molecular confirmation of cases was performed by the Pasteur Hellenic Institute. All newborns suspected of CT received treatment and were serologically and clinically followed up for one year.

**Results:**

The monthly response rate reached 100%, although only after reminders sent to 65% of the participant physicians. Sixty-three suspected CT cases were recorded by the DSN during the study period including fourteen confirmed and seven probable cases. Ten cases (47.6%) presented with symptoms at birth. Chorioretinitis was the most prominent manifestation, occurring in five symptomatic CT cases (50%). No other symptoms appeared by the end of the one year clinical follow up. No case was recorded by the existing surveillance system of the Hellenic Center of Disease Control and Prevention (HCDCP) during the same time period. Birth prevalence was estimated at 0.45, 0.51 and 0.51 per 10,000 births for 2007, 2008 and 2009 respectively. The incidence rate of symptomatic CT at birth was estimated at 0.10 cases per 10,000 births per year in Greece (for the period 2007–2009).

**Conclusion:**

The DSN for CT proved to be more sensitive than the classical notification system, easy in application and very efficient in reporting rare diseases such as CT. Similar DSNs could be used to provide useful information on other rare diseases.

## Background

Toxoplasmosis occurs worldwide and is one of the most common parasitic infections [1]. When primary infection occurs during pregnancy, the parasite can be transmitted from the mother through the placenta to the fetus. Congenital infection is usually asymptomatic, but when symptomatic it may cause neurological or visual impairment or even death [2].

Strategies for the prevention of congenital toxoplasmosis depend largely on the incidence of the disease. Several surveillance schemes have world widely been implemented for reporting CT cases in order to assess the incidence of CT. In USA, information on the CT incidence was derived from the neonatal screening program by the New England Regional Toxoplasmosis Working Group, which showed an estimated incidence ranging from 0.1 to 1 per 1000 live births in different areas [3]. In Europe, there is a high heterogeneity between countries in the surveillance of the disease. Available data on the burden of CT provided by epidemiological surveys are limited in number and validity. A survey conducted within the EUROTOXO project in 2004 among 28 European countries revealed that only 14 had a surveillance system for toxoplasmosis (congenital or not). Denmark, France, Germany, and Italy (the latter only at regional level), were the only participating European countries who had implemented a surveillance system that was specifically dedicated to CT and that was able to detect symptomatic as well as asymptomatic cases [4]. Systems which survey toxoplasmosis in the general population are of least interest because it is impossible to distinguish congenital from acquired toxoplasmosis without data on the serological status at birth [5]. Furthermore, the vast majority of acquired *Toxoplasma gondii* infections in immunocompetent individuals are benign and the proportion of asymptomatic cases is estimated to be 70% [6].

CT has been a notifiable disease in Greece since 2004 and subject to continuous data collection by the HCDCP. The health event under surveillance is toxoplasmosis (congenital or not), as defined by the European Commission decision (symptomatic toxoplasmosis cases serologically confirmed) [7].

The aim of the study was to apply a DSN for CT, in order to assess the birth rate of CT in Greece including asymptomatic cases and also to compare it to the existing notification system, which is based only on symptomatic CT.

## Methods

### Ethics and consent statement

This study was approved by the ethics committee of the Medical Faculty of the University of Thessaly. The doctors who participated in the study informed the parents about the purpose of the study and obtained verbal consent.

The network was established within the Greece-Cyprus Pediatric Surveillance Unit (GCPSU), which has been a member of the International Network of Pediatric Surveillance Units since 2002.

### Establishment of network-population under surveillance

The GCPSU supervised and managed this study over the period from April 2006 to December 2009. Specifically, in April 2006 the Unit decided to implement a new active surveillance scheme for the detection of CT cases, based on a group of selected clinicians, who were able to diagnose the disease at different stages. The Toxoplasmosis Study Group (TSG) was established including thirty clinicians of different specialties (18 pediatricians, 7 neonatologists, 3 ophthalmologists, 2 neurologists) from different geographical regions covering the whole of Greece. The network was based mostly on secondary and tertiary health care university hospital experts, as it was presumed that cases of CT from district hospitals or private clinics would most probably be referred to university hospitals for diagnosis and treatment. One person from each registered hospital was defined as the focal point of the study and was responsible for reporting any suspected case. The network was initially established in the District of Thessaly in Central Greece, under the supervision of the Laboratory of Hygiene and Epidemiology of the University of Thessaly.

Regular notification of CT cases commenced in January 2007, when the network covered almost 86% of the population of children (Districts of Thessaly, Macedonia, Thrace, Peloponnesus, Sterea Hellas and Attica), whereas by January 2008 it reached approximately 95% of the Greek pediatric population (including the Districts of Crete and Epirus). We assume the actual coverage was more than 97% of the pediatric population, as patients in remote areas (i.e. the Aegean and the Ionian Islands) are usually referred to hospitals in metropolitan cities.

### Reporting system based on active surveillance

Reporting was based on monthly cards (including zero reporting). Clinicians were supplied with monthly reporting cards and were asked to report any suspected case that met the reporting criteria. Cards were faxed or e-mailed to the Laboratory of Hygiene and Epidemiology, where data were gathered and entered into a database. The scientific coordinator of the study was responsible for identifying duplicate reports. Clinicians were reminded (by phone or e-mail) at the beginning of the next calendar month if a monthly report was not received. When a suspected case was reported, a questionnaire was sent to the reporting clinician to obtain more details about the case (including prenatal history, mother’s medical history, and laboratory and imaging results of the patient, physical examination and treatment). Personal contact details of the patients were kept on a separate sheet of the questionnaire by the coordinator to ensure protection of personal data. Samples of all patients were sent to the Pasteur Hellenic Institute, which was used as the reference laboratory for confirmation of suspected cases.

### Case definitions

Clinicians were asked to report any newborn or infant ≤12 months old (after this age IgM or IgA may reflect acquired infection) or stillbirth in whom CT was suspected.

The criteria for suspected CT included detection of *Toxoplasma*- specific IgM IgA, and IgG antibodies, or presentation of hydrocephalus, intracranial calcifications, microcephaly, retinitis, micropthalmia, unexplained lymphadenopathy and hepatosplenomegaly in infancy.

A probable case of CT was defined as a child <12 months of age with unexplained hepatosplenomegaly or lymphadenopathy or any symptom of the classical triad (hydrocephalus, intracranial calcifications and chorioretinitis) and the presence of *Toxoplasma* IgG antibodies, with maternal serological results compatible with confirmed infection during pregnancy) or any asymptomatic infant <12 months of age with high levels of *Toxoplasma* IgG (>300 IU /ml) and maternal serology compatible with *Toxoplasma* infection during pregnancy.

A confirmed case of CT (asymptomatic or not) was diagnosed either by the demonstration of *Toxoplasma* in body tissues or fluids, and/or the detection of *Toxoplasma* nucleic acid in a clinical specimen, and/or the *Toxoplasma* specific antibody response (IgM, IgG, IgA) in a newborn, and/or the persistently stable IgG *Toxoplasma* titre in an infant up to 12 months of age [8].

However, if the child has been treated continuously with sulfadiazine and pyrimethamine, the synthesis of *Toxoplasma*-specific IgG antibodies can be suppressed, and the serological confirmation of the infection cannot be made with certainty [9].

### Estimation of birth prevalence-denominators

The birth prevalence of CT (per 10,000 live births per year) for the specific study period was estimated by the number of newborns with confirmed or probable disease divided by the number of births in Greece (84,032 births during the period April-December 2006, 111,926 births in 2007, 118,302 births in 2008 and 117,933 births in 2009 respectively).

## Results

By December 2009, 1320 cards had been collected by the Unit. Response rate reached 100% during the surveillance period. On average, monthly reminders were sent to 65% of the reporting clinicians.

A total of 85 cases were reported during the study period. Specifically, 43 cases were reported by pediatricians, 39 by neonatologists, and 3 by ophthalmologists, whereas no case was initially reported by neurologists. Most reports were sent by hospitals in metropolitan areas revealing the fact that patients usually are referred to tertiary health care centres (university hospitals) for treatment and follow up. Seven of the 85 reported cases did not fulfill the inclusion criteria as they were older (3 probable CT cases presenting with chorioretinitis and 4 possible acquired toxoplasmosis cases). Another 7 cases were reported by more than one source providing information on different clinical stages o f the same patient. Finally, in 8 reported cases no further information was provided because of parents’ refusal or emmigration. A total of 22 reported cases were thus excluded from the study. The remaining 63 reported cases which were considered as suspected CT included 34 boys (54.0%), 28 girls (44.4%), and one stillborn child. Thirty-three (52.4%) patients were Greek, while 30 (47.6%) immigrants (Albanians, Russians, and Bulgarians) and local Roma. They were all born in Greek neonatal departments by mothers living the last five years in Greece and none of the mothers had travelled abroad during pregnancy.

In total, the 63 suspected CT cases included 14 confirmed and 7 probable cases. More specifically, 9 confirmed and 4 probable CT cases were reported by pediatricians, 5 confirmed and 3 probable CT cases by neonatologists (Table 1). Age-wise, the cases included 17 children aged 0–3 months and 4 children aged 4–12 months. Eight patients were Greek (38.1%), 7 were Albanian (33.3%), 3 were local Roma (14.3%), 1 from Bulgaria (4.8%) and 2 (9.5%) from the former Soviet Union. Eleven of the cases (64.7%) presented with no symptoms at birth.

**Table 1:** Classification of suspected, confirmed and probable cases according to the reporting source

	**Number of participants**	**C.T. reported**	**C.T. suspected**	**C.T. confirmed**	**C.T. probable**
**Neonatologists**	7	39	39	5	3
**Pediatricians**	18	43	24	9	4
**Ophthalmologist**	3	3	0	0	0
**Neurologists**	2	0	0	0	0
**Total**	30	85	63	14	7

All newborns were regularly followed up both clinically and serologically until the first year of age (at this point no interference from the coordinator of the study was done for the treatment and follow up by the reporting clinicians). Clinical evaluation included pediatric, neurological and ophthalmological assessment.

Among the 21 confirmed and probable cases, 7 patients (33.3%) presented a severe form of the disease, including 3 patients with bilateral chorioretinitis and intracranial calcifications, and one case each of multiple intracranial calcifications and hypertonia; hydrocephalus and bilateral chorioretinitis; microcephaly, dilatation of ventricles and intracranial calcifications; and of bilateral chorioretinitis in a small for age patient. The moderate lesions or signs included hepatosplenomegaly, rash, jaundice, hypertonia or hypotonia. The majority of cases (80.9%) were diagnosed during the first month of life (mean age at diagnosis 20 days), as a result of serological examinations of newborns born to mothers with a history of toxoplasmosis during pregnancy. The clinical follow up of children for one year did not reveal any further new symptoms.

According to the presented data, the birth prevalence of CT was approximately 0.45 cases per 10,000 births in 2007, 0.51 per 10,000 in 2008 and 0.51 per 10,000 in 2009 (Table 2). The incidence rate of symptomatic CT at birth was estimated at 0.10 cases per 10,000 births per year in Greece (for the period 2007–2009).

**Table 2:** Classification of suspected, probable and confirmed cases during the study period April 2006 to December 2009, Greece

	**Suspected cases**	**Confirmed cases**	**Probable cases**	Birth prevalence (per 10,000 live births per year) (95% CI)*
April 2006-December 2006	9	2	2	0.48** (0.13-1.22)
January 2007-ecember 2007	21	2	3	0.45 (0.15-1.04)
January 2008-December 2008	17	5	1	0.51 (0.19-1.10)
January 2009-December 2009	16	5	1	0.51 (0.19-1.11)
**Total**	**63**	**14**	**7**	

*Confidence intervals based on the Poisson distribution.

**This corresponds to a period of 9 months (April-December).

In contrast to the results of the DSN, no cases were reported by private physicians or hospital clinicians during the three year period with the notification system of the HCDCP. Looking for cases during the previous years, only two cases of symptomatic CT were reported to the HCDCP between 2004 and 2006 (provided by HCDCP).

## Discussion

The DSN for CT managed to identify fourteen confirmed cases and seven probable cases during the study period. No case was reported to the mandatory notification system of the HCDCP for the same time period. Surveillance of CT with the current system of the HCDCP has been in existence since 2004 based only on case definitions of symptomatic CT.

Notification in Greece is based strictly on the clinician. The heavy scheduled program of most doctors and their belief that surveillance is time-consuming are factors leading to defects in the reporting system. In previous studies in other countries, underreporting of diseases was attributed to ignorance about the requirements or methods of reporting, including not knowing which diseases are required to be reported, not knowing how a disease should be reported, concerns regarding confidentiality and perceptions that the list of reportable diseases is too extensive [10-13].

However, the DSN succeeded in collecting an adequate number of reports by using a small number of clinicians from various disciplines who collaborated under the same spectrum of cases of CT. A very small number of the reports were duplicated (sent both by neonatologists, pediatricians and ophthalmologists) also revealing the effectiveness of cross matching, hence reducing the possibility of missing any case. A laboratory surveillance network was set up, by the name ToxoSurv, in France in 2006 based on a network of specialized laboratories certified in prenatal and neonatal diagnosis of toxoplasmosis. The system provided more robust and reliable results due to the exhaustive process adopted for notification (all laboratories carrying out the diagnosis in France were invited to participate in the surveillance) [14]. In our study, one reference laboratory (the Hellenic Pasteur Institute) was used to carry out the confirmation of suspected cases, either by serological or PCR techniques, to avoid differences in methods used by laboratories of the selected hospitals.

Response rate was quite high throughout the period of surveillance, but required reminding through telephone communication or by emails. This system of personal contact proved to be efficient and cost-effective (involving one-person part-time job) in a small country like Greece. Both the e-mail based reminding system and the web-based reporting system proved simple, flexible and minimized paperwork.

In our study the prevalence of CT was estimated without taking into consideration the number of induced abortions or terminations of pregnancy after prenatal diagnosis of CT and this could be a reason for underestimating the true birth prevalence of CT in Greece. To overcome this limitation in the future, we believe that a network of pediatricians, neonatologists and obstetricians would be an ideal combination to study the epidemiology of uncommon birth disorders in Greece. Another reason why our data may be an underestimate of the true prevalence of CT in Greece involves administration of treatment to infected pregnant women and their neonates, which could have affected both the number of confirmed cases and the proportion of symptomatic ones.

Compared to epidemiological data in other countries, the birth prevalence of CT in Greece seems much lower than in the European countries involved in the EUROTOXO project (Bénard A, Salmi LR, Mouillet E: Systematic review on the burden of congenital toxoplasmosis in Europe, unpublished), particularly Sweden. It should be noted, however, that the EUROTOXO data were largely collected before 2000. According to the EUROTOXO results, in Austria the birth prevalence of CT was estimated at approximately 7.4 per 10,000 births [15], in Denmark at 3 per 10,000 births [16], in Germany at 13 per 10,000 births [17], in Poland at 11.3 per 10,000 births [18], in Sweden at 0.73 per 10,000 births [19], in Switzerland at 5 per 10,000 births [20] and in Italy (on a regional level) 13.8 per 10,000 newborns [21]. The incidence of symptomatic CT observed in our study was comparable to those observed in England and Ireland between 2002 and 2004 (0.16 [95% CI: 0.08-0.28] per 10,000 births) [22] and in France (0.34 [95% CI: 0.2-0.5] cases per 10,000 births) in 2007 [14].

Congenital toxoplasmosis may be characterized as a low-incidence disease in Greece, but not of low importance, due to its potentially severe complications. Epidemiological data for toxoplasmosis in general come from scarce studies throughout the country. Seroprevalence of *Toxoplasma* infection in the general Greek population ranges from 32.2% to 45% [23-25], whereas a 29.2% seroprevalence was reported in pregnant women [26] and the seroconversion rate has been estimated to be 3.3% [27]. Variations may be due to a number of reasons, including varying eating habits in different areas, different climatic conditions etc.

Ten symptomatic CT cases at birth (47% of all confirmed and probable cases) were reported during the study. Chorioretinitis was the most frequent manifestation (50% of symptomatic cases). Although there is a low incidence of primary maternal infection in Greece, the uncertain benefit of prenatal screening to a small number of women and children should not outweigh the risk of side effects from invasive diagnosis and the high cost of diagnostic methods and treatment. Studies have shown that in the absence of prenatal treatment, the frequency of chorioretinitis and cerebral lesions was higher [28].

Geographical distribution of cases was variable. The higher prevalence observed in the regions of Thessaly, Macedonia and Attica may be due to different cli matic characteristics of those areas or different dietary habits of the resident populations. Further studies are needed to elucidate the reasons for such a variable geographical distribution of CT cases.

The estimated lower birth prevalence by using the data of the DSN compared to those in other countries may be attributed to different methodological approaches and the use of different case definitions and classification systems at the time of the study in each country. Additionally, epidemiological results for other countries are based on screening programmes which have been implemented and tested for years in contrast to the results of GCPSU, which were based on an experimental pilot surveillance system. This DSN study only detected lesions evident at birth. However, the burden of CT would be better assessed by long-term follow-up of cases, as congenitally infected newborns that are asymptomatic at birth are at risk of developing ocular lesions during childhood and adolescence, leading to visual impairment. Thus, greater cohorts and long-term follow-up are required to confirm the encouraging results of the present study.

## Conclusion

A dedicated surveillance network facilitated by GCPSU proved to be a sensitive and cost-effective means for capturing newly diagnosed cases of CT. The results presented here should encourage clinicians of different specialties to actively participate in this and other study groups, allowing for the active surveillance of other rare diseases as well.

## Abbreviations

CT: Congenital Toxoplasmosis; DSN: Dedicated Surveillance Network; GCPSU: Greece Cyprus Pediatric Surveillance Unit; HCDCP: Hellenic Center of Disease Control and Prevention.

## Competing interests

The authors declare that they have no competing interests.

## Authors’ contributions

MA is responsible for the data collection and drafted the manuscript. CH developed the study design. All authors participated in developing the study protocol. All authors have read and corrected draft versions of the manuscript and approved the final manuscript.

## Pre-publication history

The pre-publication history for this paper can be accessed here:

http://www.biomedcentral.com/1471-2458/12/1019/prepub
